# One-on-one mentoring for final year medical students during the neurosurgery rotation

**DOI:** 10.1186/s12909-021-02657-0

**Published:** 2021-04-22

**Authors:** Felix Behling, Isabella Nasi-Kordhishti, Patrick Haas, Joey Sandritter, Marcos Tatagiba, Stephan Herlan

**Affiliations:** 1 Department of Neurosurgery, University Hospital Tübingen, Eberhard-Karls University Tübingen, Hoppe-Seyler Street 3, Tübingen, Germany; 2 Center for CNS Tumors, Comprehensive Cancer Center Tübingen-Stuttgart, University Hospital Tübingen, Tübingen, Germany; 3 Institute of Clinical Anatomy and Cell Analysis, Eberhard-Karls University Tübingen, Tübingen, Germany

**Keywords:** Mentoring, Final year medical school, Clinical clerkship, Neurosurgery, MiniCEX

## Abstract

**Background:**

Medical students show varying clinical practical skills when entering their final year clinical clerkship, which is the final period to acquire and improve practical skills prior to their residency. We developed a one-on-one mentoring program to allow individually tailored teaching of clinical practical skills to support final year students with varying skill sets during their neurosurgical clinical clerkship.

**Methods:**

Each participating student (*n* = 23) was paired with a mentor. At the beginning students were asked about their expectations, teaching preferences and surgical interest. Regular meetings and evaluations of clinical practical skills were scheduled every 2 weeks together with fixed rotations that could be individually adjusted. The one-on-one meetings and evaluations with the mentor gave each student the chance for individually tailored teaching. After completion of the program each student evaluated their experience.

**Results:**

The mentoring program was well received by participating students and acquisition or improvement of clinical practical skills was achieved by most students. A varying practical skill level and interest in the field of surgery was seen.

**Conclusions:**

A neurosurgical one-on-one mentoring program is well received by final year medical students and allows for individually tailored learning of clinical practical skills.

**Supplementary Information:**

The online version contains supplementary material available at 10.1186/s12909-021-02657-0.

## Background

In Germany during the final year of medical school students have the last chance to acquire and refine their clinical practical skills during mandatory clinical clerkships. Thus, student’s expectations are high in regard to prepare themselves for the final medical exam and upcoming residency programs. Different approaches to prepare medical students for clinical practical rotations have been described and integrated into medical school curricula. For example, instrument-based examinations such as ECG, ultrasound, auscultation and endoscopy can be introduced to medical students via skills labs and patient simulators [1, 2]. This way, students can experience the handling and technical aspects of instrument-based examinations and can practice in a “safe” environment. Additionally, clinical examination skills can be acquired as part of student group examination courses or further refined with patient actors [3].

However, applying clinical practical skills in daily hospital routine can be challenging for medical students [4] and some parts of it cannot be learned through simulation [5]. We have observed a marked difference among final year medical students regarding their clinical practical skills. This may be based on differences in individual interest, practical talent and dexterity as well as the educational quality of prior clinical rotations. Some medical students may also have gained specific practical skills due to part time jobs in the medical field prior or during their medical education. Especially this individual variation makes the clinical teaching of final year medical students a great challenge if a coherent educational objective is pursued. On the other hand, daily clinical practice provides limited time for teaching doctors for assessing and supporting practical skills and deficits of medical students on an individual basis [6]. This is especially difficult in a busy large university hospital where students are expected to integrate quickly in a large team of medical personnel (residents with differing grades of clinical and didactic experience, nurses, physiotherapists, specialized technicians etc.). A possibility to address this problem is a mentoring program for medical students. Its potential in a obstetrics and gynecology clerkship has been demonstrated with the result that it was well received by medical students [7].

Especially in highly subspecialized fields that are extremely demanding for residents it is notoriously challenging to integrate and train medical students properly. This is particularly true for the field of Neurosurgery. We therefore developed a one-on-one mentoring program to provide final year medical students with an individually structured rotation during their clinical clerkship in our neurosurgical department with the goal to demonstrate the feasibility and reception by medical students. Our experience and the results from the evaluation by the participating students are presented.

## Materials and methods

We have constructed a one-on-one mentoring program for final year medical students for their 8-week neurosurgical clinical clerkship. A total of 23 students took part in this program which consisted of regular assessments of clinical practical skills and evaluation meetings every 2 weeks. During the first one-on-one meeting the mentor presented the structure of the mentoring program to the mentee, which consisted of four 2-week rotations (Table 1).

**Table 1:** Rotations and miniature clinical evaluation exercises (MiniCEX) of the neurosurgical clinical clerkship within the mentoring program

	Rotation	MiniCEX
Week 1–2	Neurosurgical ward	Preoperative neurosurgical physical examination
Week 3–4	Neurosurgical ward/OR	Neurosurgical ward round
Week 5–6	Outpatient clinic/OR	Postoperative management of a neurosurgical patient
Week 7–8	Intensive care unit/management of neurosurgical emergencies	Examination of a comatose/sedated neurosurgical patient

Each student was asked about the current status regarding basic clinical practical skills, possible deficits and ways of targeted improvement. Individual teaching and instruction were offered according to each students’ need and demand. This way especially students with weaknesses regarding certain clinical practical skills were encouraged to accept help and guidance by the mentor in a safe one-on-one environment. Furthermore, each student was asked to express special interests for the opportunity acquire further clinical practical skills beyond the requirements of the clinical clerkship (e.g. lumbar drain or intracranial probe placement, advanced suturing techniques, etc.) or for an individually designed rotation plan (e.g. longer operative room rotations, a rotation with the on-call team, etc.). This way mentees with already highly developed clinical practical skills and special interest had the chance for further skill acquirement.

Scheduled one-on-one meetings took place every 2 weeks. Before each meeting the student was assessed by the mentor for certain clinical practical skills via a miniature clinical evaluation exercise (MiniCEX). The content and requirements for each examination were given to the student by the mentor at the prior meeting (Table 1). This way the mentee had 2 weeks to focus on the practical skill tested in the upcoming exam. Each exam focused on basic clinical skills that each student encountered in daily clinical practice during his 8-week neurosurgical clinical clerkship (Supplementary material 1). The respective MiniCEX sheet was used for the evaluation of the MiniCEX performance and handed out to the student after the exam together with personal feedback in a one-on-one environment to promote further skill improvement.

In all meetings further topics beyond practical skills were discussed according to the mentees demands and wishes. The mentor encouraged the mentee during each meeting to express the need to discuss other topics. This included talking about challenging interactions with patients, their next of kin or medical personal of different fields (physicians, nursing staff, physiotherapists and scrub nurses, etc.) as well as career advice and personal development feedback.

Inclusion in the mentoring project was optional and each participating student was asked to fill out evaluation forms at the beginning (*n* = 23) and after completion of the rotation (*n* = 20). Three students did not submit the final evaluation sheet. The evaluation at the beginning focused on the surgical interest, students’ expectations of the clinical clerkship and clinical practical teaching preferences. The final evaluation included the students’ assessment of the mentoring program regarding structure and contents (Supplementary material 2). All methods were carried out in accordance with relevant guidelines and regulations. Since participation and anonymous evaluation at the end of the project was voluntary, we did not obtain informed consent after consultation with the Clinical Ethics Committee of the University of Tübingen (Project number 485/2018BO2), which approved the study.

All mentors who participated in the mentoring program, had finished or were in the middle of a training course of the Center for Teaching and Learning of the University of Tübingen. Additionally, all mentors had sufficient experience in the teaching of medical students.

## Results

### Evaluation at the beginning of the mentoring program

All participating students filled out the evaluation form at the beginning of the mentoring program (*n* = 23). When asked to list clinical practical skills they wished to acquire during the final year clinical clerkships 26% (6/23) indicated basic skills like drawing blood, physical examination techniques and general ward work, while 70% (16/23) listed more advanced skills like sonography, lumbar puncture and placing central lines. Thirty-five percent expressed the wish to acquire suturing techniques (8/23) and only 9% had the desire to assist in operations or even acquire basic neurosurgical skills like the placement of an intracranial pressure probe (2/23). Eighty-seven percent answered that individually tailored instructions are an important aspect when learning clinical practical skills (20/23). Ninety-six percent preferred a one-on-one teaching environment for acquiring clinical practical skills (22/23), while 26 % regarded group teaching of practical skills as important (6/23). An interest in the field of surgery was expressed by 52 % (12/23) while 30 % disagreed with that statement (7/23) and 17 % (4/23) had a neutral surgical interest. The majority of the participating students (87%, 20/23) stated to already have decided which medical field to enter after graduation. Learning clinical practical skills beyond the requirements of the final year clerkships was regarded as important by 83 % (19/23) while 17 % answered neutral or with disagreement (4/23). Sixty-five percent (15/23) agreed with the statement that the acquisition of such competencies is important for their individual development as a physician while one student strongly disagreed (4%) and twenty-two had a neutral opinion (5/23).

**Fig. 1:**
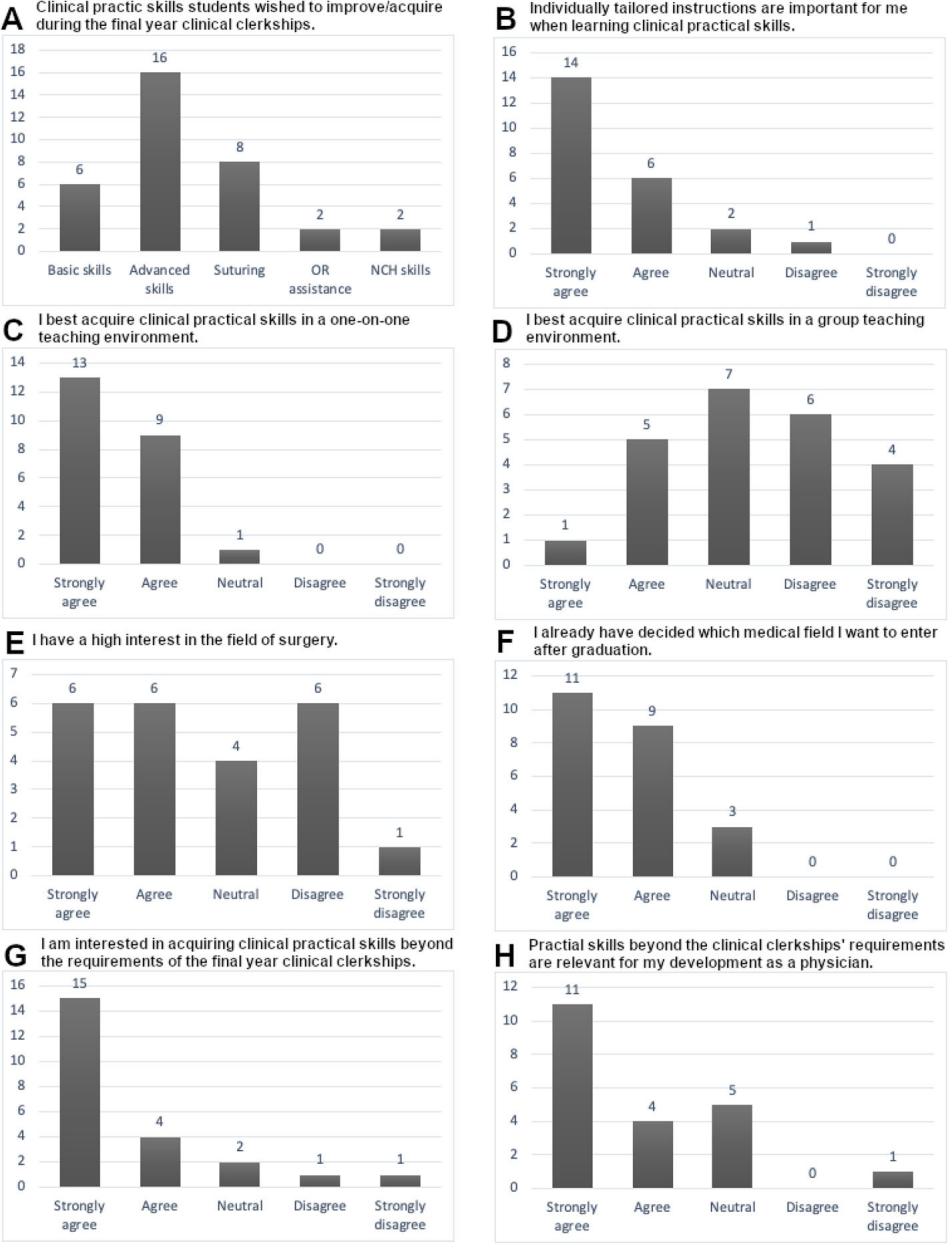
Results of the evaluation at the beginning of the mentoring program

Details of the student evaluation at the beginning of the mentoring program are presented in Fig. 1.

### Evaluation after completion of the mentoring program

Eighty-seven percent of the participants submitted the final evaluation sheet (20/23). All were satisfied with the organization and the quality of the regular one-on-one meetings with the mentor. The organization of the MiniCEX was also rated as good or very good by all students. Ninety-five percent (19/20) stated that the MiniCEX were a helpful element for acquiring or improving clinical practical skills while one student expressed a neutral opinion. Seventy-five percent expressed to have acquired or improved clinical practical skill during the mentoring program (15/20). Two students disagreed with this statement (10%). Ninety-five percent rated the mentoring program as good or very good (19/20) and 80 % would recommend the program to other medical students while 20 % gave a neutral response (4/20).

**Fig. 2:**
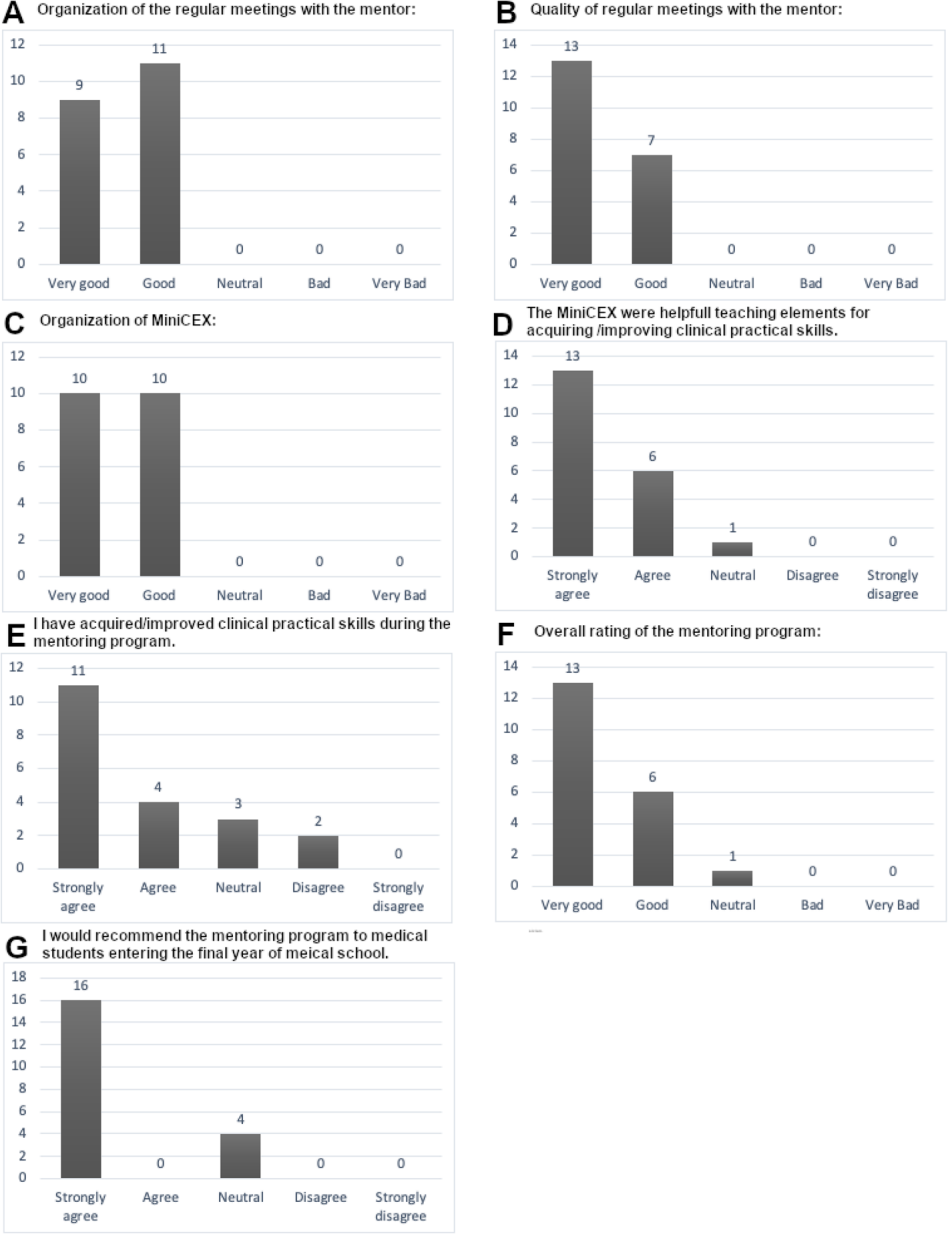
Results of the evaluation after completion of the mentoring program

Details of the final evaluation are represented in Fig. 2.

## Discussion

Deficits in clinical practical skills of final year medical students in Germany have been described in a study by Krautter et al. in 2015 [4]. We have also observed pronounced differences in clinical practical competencies among final year medical students which led us to design the one-on-one mentoring program. A similar mentoring initiative for Obstetrics and Gynecology clerkships has been reported and was well received by participating students. While the emphasis was laid on weekly meetings discuss variable topics according to the mentees needs and wishes [7], we have extended the focus of our mentoring program to individualized clinical practical skill development and individual rotation options.

Despite the opportunity for students to get familiarized with examination techniques and small procedures in skills labs and simulated settings, the reality of daily hospital routine represents a new challenge to medical students and can be overwhelming [8]. The skill set that medical students bring with them when entering their final year clerkships is quite variable based on prior chosen rotations and their teaching quality as well as interest in clinical practical skills and their individual dexterity. Additionally, students may rotate to our department at the beginning, in the middle or at the end of their final year clinical clerkships. During the mentoring program we confirmed that there was a high variability in clinical practical competencies. While some students expressed the need to acquire basic skills like drawing blood or physical examination techniques, others were already quite competent and routinely performed these tasks from the beginning of the program. A few students showed a high interest in acquiring more specific basic neurosurgical skills. This underlines the need to approach final year medical students individually regarding clinical practical skill acquirement and improvement.

The one-on-one setting was regarded as a strong point of the program by the majority of the students. Most students expressed to best learn practical skills in a one-on-one teaching environment than in a group session. We believe that this is based on the creation of a safe one-on-one environment that gives each student the chance to address clinical practical weaknesses. It also allows for a more specific feedback, which has the potential to guide further improvement individually.

Overall, the program had a high approval rate of 95%, although only 52% expressed an interest in the field of surgery at the beginning of the program. The structure and organization were also well received by the participating students. The organization and quality of the feedback and evaluation meetings were graded as ‘good’ or ‘very good’ by all participants. It has been reported that medical students regard it as important to have time to perform examinations independently and also under supervision with appropriate feedback in order to have good learning experiences during clinical clerkships [9]. In regard to this, our mentoring program offered a good learning structure with regular meetings paired with short clinical examinations (MiniCEX) that the students had to prepare for during the prior 2 weeks during their integration into daily hospital routine. The MiniCEX were an ideal tool to give medical students a clear objective of each 2-week rotation and also allowed for specific feedback based on the individual clinical practical skill set of each student. This is reflected by the high approval rate of MiniCEX as a helpful learning tool by 95% of the students.

The interest in surgical and neurosurgical practical skills among final year medical students is very different as shown by our data. The majority of final year medical students revealed no interest in OR assistance or acquiring neurosurgical skills. It is also worth mentioning that the majority of students had already decided on a specialty they wanted to enter after graduation. It is clear that neurosurgery is a very specific clinical rotation and most medical students are assigned to our department as part of their mandatory surgical rotation, while few chose the rotation based on special interest. As a highly specialized surgical field, it is challenging for a neurosurgical department to provide an encouraging and motivating environment for medical students with little or no interest in the field of surgery. It is another strength of our mentoring program to give students with low surgical interest an individually tailored learning experience. One important characteristic was the opportunity for rotations based on students’ special interest (e.g. longer rotation on the intensive care unit or the outpatient clinic instead of the OR). On the other hand, students with a high surgical interest or even with the wish to pursue a surgical career, had the chance to acquire clinical practical skills beyond the final year clerkship requirements that gave them an insight into what to expect during upcoming residencies. It is well known that experiences in surgical rotations have an impact on students’ career choices [10] which stresses the need for medical students to get a deep clinical experience during each of their rotations and clerkships. A mentoring program is an effective format to offer this to students with varying interest and clinical skill sets.

## Conclusion

A neurosurgical one-on-one mentoring program is well received by final year medical students and allows for individually tailored learning of clinical practical skills.

## Supplementary Information

**Additional file 1.**

**Additional file 2.**

## Data Availability

The dataset of the study is available from the corresponding author on reasonable request.

## Abbreviations

ECG: Electrocardiography
MiniCEX: Mini Clinical Evaluation Exercise
