# Supplementary material for: One-on-one mentoring for final year medical students during the neurosurgery rotation
Source: BMC Med Educ. 2021 Apr 22;21:229. doi: 10.1186/s12909-021-02657-0 (PMC8061075; doi:10.1186/s12909-021-02657-0)
Supplement: Supplementary file 1 — Additional file 1. [file 12909_2021_2657_MOESM1_ESM.pdf]

Neurochirurgie
Uniklinik Tübingen

Mentoring im
Praktischen Jahr

Clinical Evaluation Exercise (MiniCEX)

1: Preoperative neurosurgical physical examination

Name of student: ____

Name of mentor: ____

	Not done	Done partially or incorrectly	Done correctly
Evaluates alertness and orientation	O	O	O
Evaluates handedness	O	O	O
Asks about current mood	O	O	O
Examines cranial nerves	O	O	O
Examines motor strengths	O	O	O
Evaluates sensitivity	O	O	O
Examines muscle stretch relfexes	O	O	O
Evaluates coordination of all four extremities	O	O	O
Performs gait examination	O	O	O
Performs disease specific examinations (Lasegue, Trendelenburg, rigor, tremor, etc.)	O	O	O
Evaluates skin area of planned surgery	O	O	O
Correct interpretation of examination findings	O	O	O
Structured examination	O	O	O
Professional demeanor	O	O	O
Empathy	O	O	O
Explains examination steps in patient appropriate terms	O	O	O
Gentle examination technique	O	O	O

Cares for patient comfort	O	O	O
	**Requirements not met**	**Satisfactory performance**	**Exceptional performance**
**Overall performance**	**O**	**O**	**O**

What did the student do especially good?

What can be improved?

Date and signature of mentor:

Neurochirurgie
Uniklinik Tübingen

Mentoring im
Praktischen Jahr

Clinical Evaluation Exercise (MiniCEX)

2: Neurosurgical ward round

Name of student: ____

Name of mentor: ____

	Not done	Done partially or incorrectly	Done correctly
Introduction with name and function	O	O	O
Asks about current condition	O	O	O
Evaluates degree of independence regarding mobilization, personal hygiene and food intake.	O	O	O
Assesses disease and treatment specific problems	O	O	O
Evaluates current pain with individual pain scale	O	O	O
Assessment of wound(s)	O	O	O
Performs a targeted disease-specific neurologic examination	O	O	O
Explains new findings and examination results (lab, imaging, etc)	O	O	O
Outlines the further course	O	O	O
Cares for patient-oriented setting	O	O	O
Structured performance	O	O	O
Professional demeanor	O	O	O
Empathy	O	O	O
Patient appropriate language/terms	O	O	O
Permits questions by patient	O	O	O

	Requirements not met	Satisfactory performance	Exceptional performance
**Overall performance**	**O**	**O**	**O**

What did the student do especially good?

What can be improved?

Date and signature of mentor:

Neurochirurgie
Uniklinik Tübingen

Mentoring im
Praktischen Jahr

Clinical Evaluation Exercise (MiniCEX)

3: Postoperative management of a neurosurgical patient

Name of student: ____

Name of mentor: ____

	Not done	Done partially or incorrectly	Done correctly
Evaluates alertness and orientation	O	O	O
Evaluates current pain with individual pain scale	O	O	O
Addresses current complaints	O	O	O
Performs a targeted disease- and surgery-specific neurologic examination	O	O	O
Examines drain (if present)	O	O	O
Explains operative course and findings	O	O	O
Outlines further course	O	O	O
Prescribes postoperative Medication if necessary	O	O	O
Correct assessment of the current medical situation	O	O	O
Structured performance	O	O	O
Professional demeanor	O	O	O
Empathy	O	O	O
Patient appropriate language/terms	O	O	O
Permits questions by patient	O	O	O

	**Requirements not met**	**Satisfactory performance**	**Exceptional performance**

**Overall performance**	**O**	**O**	**O**

What did the student do especially good?

What can be improved?

Date and signature of mentor:

Clinical Evaluation Exercise (MiniCEX)

4: Examination of a comatose/sedated neurosurgical patient

Name of student: ____

Name of mentor: ____

	Not done	Done partially or incorrectly	Done correctly
Examines alertness	O	O	O
Examines protective reflexes	O	O	O
Examines motor function (if possibe)	O	O	O
Examines pupillary reflexes	O	O	O
Evaluates ICP and EVD drain (if applicable)	O	O	O
Assessment of wound(s)	O	O	O
Cardiac examination	O	O	O
Pulmonary examination	O	O	O
Abdominal examination	O	O	O
Evaluation of the skin and pulses	O	O	O
Views and interprets monitoring	O	O	O
Views and interprets current laboratory results	O	O	O
Views and interprets current microbiology results	O	O	O
Views and interprets current radiographic examinations	O	O	O
Correct interpretation of current status and examination findings	O	O	O
Correct assessment of the current medical situation	O	O	O
Generates a treatment plan	O	O	O

Structured performance	O	O	O
Professional demeanor	O	O	O
Empathy	O	O	O
Patient appropriate language/terms	O	O	O

	**Requirements not met**	**Satisfactory performance**	**Exceptional performance**
**Overall performance**	**O**	**O**	**O**

What did the student do especially good?

What can be improved?

Date and signature of mentor:
