# Supplementary material for: One-on-one mentoring for final year medical students during the neurosurgery rotation
Source: BMC Med Educ. 2021 Apr 22;21:229. doi: 10.1186/s12909-021-02657-0 (PMC8061075; doi:10.1186/s12909-021-02657-0)
Supplement: Supplementary file 2 — Additional file 2. [file 12909_2021_2657_MOESM2_ESM.pdf]

Neurochirurgie
Uniklinik Tübingen

Mentoring im
Praktischen Jahr

Evaluation at the beginning of the mentoring program

This anonymous questionnaire evaluates the clinical practical training of final year medical students.The data will be used to improve the training and teaching quality. Therefore, please provide straight and honest answers. Note that there are no “correct” or “wrong” answers. The evaluation of all questionnaires will be done at the end of your clinical rotation so any statements or answers you give cannot impact your clinical rotation. We plan to present the results of the evaluation of the mentoring program in a scientific journal. The participation in the mentoring program and the evaluation is optional.

1. Did you work in the field of medicine prior to entering medical school?
   If yes, please describe the occupation.

   ____

   ____

   ____

   ____

2. Did you work in the field of medicine during medical school?
   If yes, please describe the occupation.

   ____

   ____

   ____

   ____

3. In what specialties did you do your clinical rotations?

____

____

____

____

4. I expect to acquire clinical practical skills during my final year clinical clerkships (e.g., drawing blood, placing IVs, physical examination, lumbar puncture, etc.).

   strongly agree 1 2 3 4 5 strongly disagree

5. I am especially interested in acquiring the following clinical practical skills during my final year clinical clerkships.

   1. ____
   2. ____
   3. ____
   4. ____
   5. ____

6. For me individual instruction is important for acquiring clinical practical skills.

   strongly agree 1 2 3 4 5 strongly disagree

7. I best acquire clinical practical skills in a group environment.

   strongly agree 1 2 3 4 5 strongly disagree

8. I best acquire clinical practical skills in a one-on-one environment.

   strongly agree 1 2 3 4 5 strongly disagree

9. How do you describe your interest in the field of surgery?

   very high 1 2 3 4 5 very low

10. Do you already know which specialty you want to enter after graduation?

very certain 1 2 3 4 5 very uncertain

11. Do you plan to enter a surgical residency program?
    A) Yes.
    B) No.
    C) Possibly.
    D) Undecided.

12. Which specialties do you consider after graduation?

    1. ____

    2. ____

    3. ____

13. I am interested in acquiring clinical practical skills that go beyond the requirements of the final year clinical clerkships.

strongly agree 1 2 3 4 5 strongly disagree

14. The acquisitiom of clinical practical skills that go beyond the requirements of the final year clinical clerkships are **not** relevant for my further development as a physician.

strongly agree 1 2 3 4 5 strongly disagree

Neurochirurgie
Uniklinik Tübingen

Mentoring im
Praktischen Jahr

Evaluation at the end of the mentoring program

This anonymous questionnaire evaluates the clinical practical training of final year medical students. The data will be used to improve the training and teaching quality. Therefore, please provide straight and honest answers. Note that there are no “correct” or “wrong” answers. The evaluation of all questionnaires will be done at the end of your clinical rotation so any statements or answers you give cannot impact your clinical rotation. We plan to present the results of the evaluation of the mentoring program in a scientific journal. The participation in the mentoring program and the evaluation is optional.

1. How do you describe your interest in the field of surgery?

   very high 1 2 3 4 5 very low

2. How do you describe your interest in the field of neurosurgery?

   very high 1 2 3 4 5 very low

3. Do you plan to enter a surgical residency program?
   E) Yes.
   F) No.
   G) Possibly.
   H) Undecided.

4. Do you already know which specialty you want to enter after graduation?

   very certain 1 2 3 4 5 very uncertain

5. Which specialties do you consider after graduation?

   1. ____

   2. ____

   3. ____

6. I acquired new clinical practical skills during the last 8 weeks.

   strongly agree 1 2 3 4 5 strongly disagree

7. How do you grade your overall experience with the teaching program *Mentoring for final year medical students in neurosurgery*?

   very good 1 2 3 4 5 very bad

8. How was the quality of the regular meetings?

   very good 1 2 3 4 5 very bad

9. How do you rate the organization of the regular meetings?

   very good 1 2 3 4 5 very bad

10. How do you rate the frequency of the regular meetings?

    A) Much too frequent.
    B) Too frequent.
    C) Just right.
    D) Too rare.
    E) Much too rare.

11. How do you rate the duration of the regular meetings?

    A) Way too long.
    B) Too long.
    C) Just right.
    D) Too short.
    E) Way too short.

12. How do you rate the organization of the MiniCEX?

    very good 1 2 3 4 5 very bad

13. Did you find the MiniCEX helpful for the acquisition of clinical practical skills?

strongly agree 1 2 3 4 5 strongly disagree

14. What aspects of the mentoring program did you especially like?

____

____

____

____

15. What aspects of the mentoring program can be improved?

____

____

____

____

16. Would you recommend the mentoring program to others?

A) Yes.
B) No.
C) Only for students with special interest.
D) Undecided.
